# Choice of inhaler device and its disposal have a significant impact on the environment

**DOI:** 10.36416/1806-3756/e20240092

**Published:** 2024-05-08

**Authors:** José Eduardo Delfini Cançado, Omar S Usmani

**Affiliations:** 1. Faculdade de Ciências Médicas, Santa Casa de Misericórdia de São Paulo, São Paulo (SP) Brasil.; 2. National Heart and Lung Institute, Imperial College, London, United Kingdom.

The delivery of therapeutic vapors and aerosols through inhalation has been used for thousands of years in various cultures. The inhalation of Datura stramonium, a leafy flowering plant (*Hyoscyamus niger*), whose therapeutic properties are attributed to tropane alkaloids, including atropine, for treatment of asthma, comes from circa 2000 BC with early traditional Ayurvedic medicine.^1^ The most prominent ancient form of respiratory drug delivery was the smoking of opium for recreational and therapeutic purposes including analgesia, and treatment of diarrhea and of severe cough, with one of the earliest known references dating back to 1100 BC in China.^2^ It is clearly recognized that, in comparison with oral or parenteral formulations, the inhaled route allows the therapeutic drug to be directly delivered topically to the airways leading to quicker local efficacy within the lungs, using lower therapeutic doses, and minimizing their systemic effects.^3^

**Figure 1:**
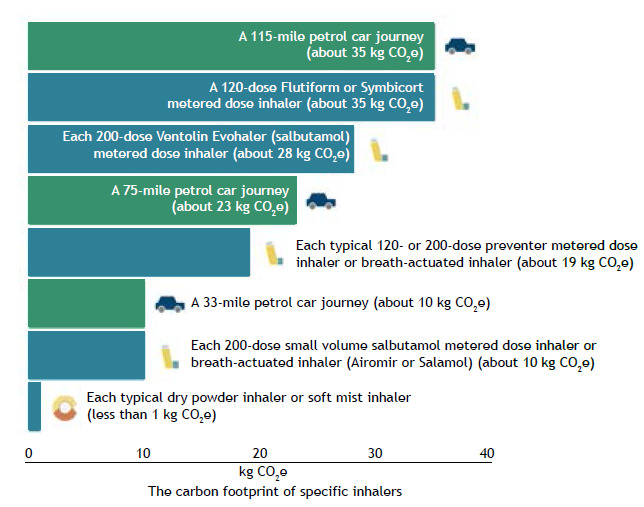
The carbon footprint of medicines and inhaler devices. Based on the National Institute for Health and Care.^5^

In many respects, the introduction of the pressurized metered dose inhaler (pMDI) in 1956 marked the beginning of the modern pharmaceutical aerosol industry, when Riker Laboratories Inc. (becoming 3M Drug Delivery Systems) introduced the pMDI. The pMDI was the first truly portable and convenient inhaler that delivered drugs to the lungs effectively, and quickly gained widespread acceptance.^4^ When originally developed, pMDIs utilized chlorofluorocarbon (CFC) propellants, but they have an effect on depleting the stratospheric ozone layer. So, in the 1990s, the Montreal Protocol led to the phasing out of ozone-damaging CFCs in inhalers. The replacement propellants were hydrofluorocarbons (HFCs). Unlike CFCs, HFCs are not ozone-depleting substances, but they are recognized as greenhouse gases that have a high global warming potential (GWP). The current HFCs in pMDIs are hydrofluoroalkane (HFA)-134a and HFA-227ea, which are 1,000-3,000 times more potent than carbon dioxide and can persist in the atmosphere for 14 years, contributing to worsening climate change. The carbon footprint from 1 pMDI (200 doses) is estimated to be equivalent to a 290-km automobile ride. Figure 1 shows the carbon footprint of different medications and inhaler devices.^5^

According to the IQVIA database (Durham, NC, USA), in the preceding 12 months until June of 2019, there were over 480 million pMDI packs prescribed, equating to 2,400 doses taken every second across the world.^6^ In Brazil, the number of pMDI short-acting β_2_ agonist (SABA) units sold has been increasing, from 24,849,295 units in 2019 to 31,156,295 units in 2023; a growth of 25.4%.^7^ With regard to the existing propellants, there is now development for transitioning to newer propellants, such as HFA-152a that has a lower GWP, and pharmaceutical companies have committed to launch newer pMDIs with this propellant by 2025-6.

Dry powder inhaler (DPI) devices do not contain greenhouse gas propellants and have a lower GWP when compared with pMDIs (Figure 1). However, DPIs are dependent on the inspiratory effort of the patient to effectively activate the dry powder to be inhaled into the lungs, and it has been shown that many patients with asthma and COPD have suboptimal inspiratory effort. Indeed, poor inspiratory effort is recognized in the young, in the elderly, and in patients with an acute exacerbation of asthma or COPD, when DPIs may not be effective. Globally, DPIs represent only 3% of the doses of SABAs, which are the mainstay of reliever medication.^6^ ^)^ Of critical importance, DPIs are not free from having an impact on planetary health due to their plastic content. Indeed, in the whole lifecycle assessment of pMDIs and DPIs, DPIs have a greater adverse effect on marine ecology through their plastic content. Since there is a global policy to curtail the effect of plastics on the environment, we must be careful in our choice of inhalers. The costs of DPIs are greater than those of pMDIs in Brazil. Soft mist inhalers are small portable devices, which are an additional class of inhalers that produce aerosols of breathable diameter from aqueous formulations. They are more environmentally friendly (Figure 1), but are currently more expensive than pMDIs and DPIs.^8^

Another important consideration is the recycling of inhalers. Of the estimated 35 million inhalers prescribed in the UK every year, only about 0.5% is recycled appropriately. Thus, millions of inhalers end up in landfills every year, where DPIs do not only significantly contribute to plastic waste, but also pMDIs release residual HFCs into the atmosphere over time.^9^

In clinical practice, the choice of drug and its dosage, treatment strategy, and inhalation device are crucial to control and prevent asthma and COPD exacerbations. Healthcare professionals, patient organizations, and the pharmaceutical industry should take the lead in health policies to provide environmentally healthier alternatives. Indeed, the greenest inhaler is the one that the patient can use (inhaler technique), will use (inhaler adherence), and has been taught how to use it properly (inhaler mastery) in order to mitigate these planet-warming greenhouse gas emissions. Ideally, prescribing physicians should educate their patients to discard used inhalation devices at pharmacies. If disposed of in regular waste, they can contaminate soil, water, and the atmosphere. However, effective recycling requires investment and policies at the governmental level to support pharmacies with appropriate equipment and clear pathways of disassembling the various parts of inhalers and their safe disposal at factories. Decree No. 10,388,^10^ published by the President of the Republic of Brazil on June 5th, 2020, regulated the reverse logistics system for expired or unused household medications for human use, both industrially manufactured and compounded, as well as their packaging. These should be disposed of at pharmacies, where they will later be collected and sent for environmentally safe disposal. In this regard, the Brazilian Thoracic Society is conducting a campaign with the aim of educating doctors, other healthcare professionals, and patients about the importance of correct inhalation treatment for prevalent diseases such as asthma and COPD, as well as proper disposal of inhalation devices at pharmacies.

It is time to put the brakes on greenhouse gas emissions and global warming. Choose the better inhaler device for each patient and teach them about the correct disposal. Let’s do it now!
